# Co-payment for Unfunded Additional Care in Publicly Funded Healthcare Systems: Ethical Issues

**DOI:** 10.1007/s11673-019-09924-2

**Published:** 2019-06-24

**Authors:** Joakim Färdow, Linus Broström, Mats Johansson

**Affiliations:** Medical ethics, Department of Clinical Sciences Lund, Lund university, 221 84 Lund, Sweden

**Keywords:** Prioritization, Justice, Co-payment, Health care needs

## Abstract

The burdens of resource constraints in publicly funded healthcare systems urge decision makers in countries like Sweden, Norway and the UK to find new financial solutions. One proposal that has been put forward is co-payment—a financial model where some treatment or care is made available to patients who are willing and able to pay the costs that exceed the available alternatives fully covered by public means. Co-payment of this sort has been associated with various ethical concerns. These range from worries that it has a negative impact on patients' wellbeing and on health care institutions, to fears that co-payment is in conflict with core values of publicly funded health care systems. This article provides an overview of the main ethical issues associated with co-payment, and ethical arguments both in support of and against it will be presented and analyzed.

## Introduction

Priorities and the rationing of resources in healthcare are discussed continuously, especially in countries where the share of public funding is high (Arvidsson 2013; Gustavsson 2017; Sandman 2017). The growing financial pressures on public welfare systems have encouraged decision-makers in countries like Sweden, Norway, the UK and Canada to consider new financial solutions for the public health care sector (Heath 2003; Godlee 2017; Mossialos et al. 2018). Lately a trend has emerged in these settings of introducing, or at least considering introducing, solutions according to which patients would be given the opportunity to pay part of the total cost of treatment, or other healthcare services, that exceeds the spending thresholds being applied within the publicly funded system. Such a solution would respect the limited public resources, while at the same time making certain healthcare options available to patients that otherwise would not be so. (It may or may not coexist with healthcare services that are fully privately funded.) In what follows, this financial model will be referred to as “co-payment”.^1^

Growing financial pressure on public welfare systems has no doubt played a role in ensuring support of the co-payment approach. Another contributory factor might have been the increased privatization of sectors and services normally funded and run by public means. Whatever *explains* the current interest in co-payment, however, need not serve as a *justification* of such a solution. Co-payment is associated with various ethical concerns (Smer 2015; Richards 2008). These range from worries that it has a negative impact on patients’ wellbeing, and on the operation of healthcare institutions, to fears that it is in conflict with core values of publicly funded healthcare systems. Whether co-payment can mitigate the problem of scarce resources is a complex matter, as evidenced by the fact that there are a number of arguments both in favour of and against allowing and/or facilitating it.

The aim of this article is to provide an overview of the main ethical issues raised by co-payment solutions, and along the way to make key points which, in our view, deserve more attention than they have hitherto received. The ethical issues surrounding co-payment need to be addressed before an informed decision can be made about whether or not to prohibit, allow, or even encourage (or otherwise facilitate) its use in public healthcare. For reasons we shall explain, however, empirical research on the actual impacts of co-payment initiatives would also have to be conducted, in order for the advantages and disadvantages of such solutions to be known. Consequently, we are not yet in a position to draw any final conclusions about the appropriateness of this model.

The general outline of this article is as follows. In the next section, a few examples will be provided of the kinds of co-payment solutions discussed in the literature, and how they differ from some other systems. In the section following this, the main ethical arguments that have been, or could be, taken to support co-payment models will be outlined. After that, various reasons for resisting such models will be presented. Here it will become evident that some problems are highly dependent on societal values, which makes it even more difficult to say something universal about the co-payment model. The article ends with some brief reflections on the lessons learned, together with some suggestions about how to proceed in matters that concern the co-payment approach.

## Co-payment: What Is It, and How Does It Work?

To illustrate the co-payment solution, imagine that an individual, P, seeks healthcare in the publicly funded healthcare system. P is, at some stage and in some way, informed that there is a choice to be made between a fully funded alternative and a more expensive one. The more expensive option may consist of a number of different things, but in the typical case it will be an extra, or more expensive, drug or device. It may promise to meet more of P’s healthcare needs, or to meet them with fewer side effects, or to satisfy them more reliably, or perhaps more rapidly. What P gets, in the way of an add-on, need not be directly or in any way related to health. It could, for example, be something with aesthetic value to P, as would be the case if P were to pay additional cost to secure a newer wheelchair, or one in a particular colour. Whatever the more expensive alternative consists of, however, co-payment will typically give P access to an add-on that (at least) many patients would agree is an additional benefit, whether that benefit is conceived of in terms of P’s health (subjective or objective) or in terms of something else that P values. The additional cost may be paid directly by P, or via P’s supplementary healthcare insurance, or through some other source such as P’s employer.

Co-payment can be implemented in many different ways. It can be fully integrated in the otherwise publicly funded healthcare system, with a public healthcare provider administrating the relevant additional care. It can also be based on, for example, the use of vouchers that can be used to purchase treatment or devices from third parties. And in the latter case, the public healthcare organization could take varying degrees of responsibility for the required coordination and for ensuring that patients will ultimately get what they additionally pay for. While in this paper co-payment will mainly be assessed as a financial solution, it should be noted that co-paid solutions will normally involve some administrative burden and responsibility for the public health organization, and that full discussion of the pros and cons of co-payment will for that reason inevitably touch upon organizational issues too. Some perceived challenges for co-payment are likely to be associated with the way it is organized, while others are of a more principled nature, something which we will return to below.

Co-payment is merely *one* way in which the relationship between public and private financing of health care can be structured. Privately funded healthcare providers working in parallel with the public system is another way of structuring this relationship, and group-based or sectorial systems can also be devised.^2^ These mixed-funding solutions may give rise to separate ethical issues (Schmidt 2007; Lesén et al. 2013; Sinnott et al. 2013; Wang et al. 2011). In what follows, however, we will only address issues linked with the kind of co-payment characterized above.

What kinds of treatment are likely to be provided under co-payment in healthcare systems, like those in the Scandinavian countries and the UK, which are, in the main, publicly funded? So far, developments in Sweden have been fairly modest and include co-payment for hearing aids with special properties and for intraocular lenses with special properties implanted during cataract surgery. In the future, however, co-payment options could emerge in almost any area of healthcare where there are pharmaceuticals, medical aids or supplies not fully covered by public funds. Devices for the monitoring and treatment of diabetes could, for example, be co-paid for, which is something that has been evaluated and discussed previously, albeit in respects other than those addressed here (Yoo et al. 2016).

In the UK, the debate over co-payment has focused on pharmaceuticals. New drugs are developed continually by the pharmaceutical industry. Although some offer advantages over prevailing treatment options, they may be expensive. In the treatment of cancer, new drugs may prolong life, or have fewer, or less serious, side effects than existing treatment options. The question whether co-payment should play this kind of role surfaced fairly recently in Norway.^3^ Looking at the treatment of chronic diseases other than cancer, pharmacological therapies where co-payment might be considered include medicines for hepatitis C and for the kidney disease Atypical Hemolytic Uremic Syndrome (aHUS). Another area where co-payment is likely to be further developed is in cases, like those mentioned above, where intraocular premium lenses are implanted during cataract surgery (Smer 2015; Anell 2014).

In theory, co-payment solutions could be introduced for anything for which there is some demand. This could include healthcare measures which have never been offered within the publicly funded system (e.g. multifocal intraocular lenses and certain devices for monitoring diabetes), or those which, for various reasons, have been removed from the publicly funded system (including expensive drugs for cancer, hepatitis C and some rare diseases). And it need not, as already suggested, involve treatment or care, in the usual sense, but could, for example, involve getting a single room on a hospital ward. It is unlikely that co-payment will be applied in all areas of healthcare, however. Although it is difficult to make precise predictions as to where the lines will be drawn, it is likely that co-payment will enter areas where the publicly funded system “falls short” in offering a commonly preferred alternative, and where there is real prospect of profit for those who develop or manufacture the drugs or other health care products suitable for additional care.

## Considerations in Favour of Co-payment

The co-payment model may or may not be welcomed by patients, but as patient demand is not in itself a strong reason for the public healthcare system to introduce this or any other model, independent arguments are called for. What, then, could speak in favour of co-payment? Three lines of argument stand out, focusing on improved health, greater patient autonomy or freedom of choice, and better patient safety.

### Health Benefits

If a treatment is considered too expensive in relation to its positive effects, it ought not be included in a publicly funded package, even if it is expected to benefit patients. This is why co-payment might have the *potential* to generate positive outcomes. Under the co-payment model, patients are able to purchase drugs or devices that would otherwise not be available to them —drugs and devices that are expected to benefit them in some way. If co-payment is not an option, some patients will be left worse off than they need to be; or so the argument goes. The cost, to the patient, of a co-payment package might be considerably lower than the outlay for the same procedure within an entirely privately funded treatment plan, which would be the only other way for the patient to access the procedure in question. Second, co-payment would make the relevant benefit available without (it is assumed) the taxpayer-funded system being burdened in any way, since the additional cost of the co-paid intervention would be met by sources outside the public sector. As a result, the public healthcare system would not need to breach agreements it has reached on spending priorities.

The success of co-payment in actually generating health benefits will obviously depend on a variety of factors: In what domains will it be implemented? What will the patients’ options be? How expensive will these options be? The point at issue is not whether or not we can imagine positive effects, as this is surely possible, but whether the co-payment model will work as intended in actual settings.

Also of relevance is the question whether patients will be encouraged to utilize co-payment options in beneficial ways that promote their health. The distinction between permitting co-payment and encouraging, or otherwise facilitating, it comes into play here. The relevant health benefits, again depending on particulars, are more likely to be secured if co-payment options are not only offered but promoted. In particular, the public healthcare system may need to be involved organizationally, to an extent, if co-payment options are actually to be taken up by patients and not be merely “available” under the relevant regulations.

Even on the assumption that the expected positive outcomes will materialize and provide at least *a* reason for allowing co-payment in the publicly funded healthcare sector, it does not follow that co-payment is justified all things considered. Co-payment might be viewed as inconsistent with core values of publicly funded healthcare, and the net outcome of co-payment could still turn out to be negative, since the model may, for example, negatively affect other interests at stake. We shall examine below what those side effects might be. But, while there is an obvious sense in which this cannot be settled a priori, and needs to be assessed empirically, it can be said in advance that the effects of co-payment will depend, to an extent, on what efforts society makes to realise the desired health benefits, and to minimize any negative side effects. In that sense, the co-payment model must be evaluated, as a package, together with society’s ambition and specific plans to secure the right outcomes. Differently put, it is simply not possible to assess the model’s potential and risks without simultaneously making assumptions about what will be done in order to maximize the chance of achieving the goals set.

### Freedom of Choice

By definition, co-payment will increase the range of options available to patients, since it will introduce alternatives that are not open to patients within the publicly funded healthcare system. Several commentators regard this feature as a major argument in favour of the model (Richards 2008; Smer 2015). The introduction of new options, and thus greater freedom of choice, has been seen as something which, at least potentially, promotes patient autonomy (Richards 2008; Oliver 2008).

Various reasons can be given for the view that more freedom of choice is a good thing. One of the most straightforward is that people typically *feel good about* being able to choose and, conversely, *feel frustrated when they are* being barred from a choice between alternatives that could have been offered to them. Whether this kind of hedonistic argument is inherently sound has been queried (Schwartz 2016), but it obviously relies on empirical assumptions that might very well turn out to be false when they are applied in the particular context of healthcare. For one thing, the outcome will arguably depend on what patients know, or believe, about what options are open, or not open, to them. This suggests that society needs not only to allow for co-payment, but to make serious efforts to make patients aware of their expanded freedom of choice. (The question also arises about the duties of healthcare professionals: should or should they not inform patients about existing interventions that are not open to them? (Bratt 2015).) In this sense there is a cognitive component to the hedonistic argument. However, it is not evident what role such awareness of co-payment options will have for the patient’s wellbeing. Even if it is true that some patients will enjoy being able to choose, others might react less favourably: co-payment for additions may highlight things those patients cannot have. This, if anything, is likely to make them feel bad rather than good. Feelings of missed opportunity (and injustice) might arise. It is safe to say, in other words, that the relation between happiness and being given greater freedom of choice is complex. Also, even on the assumption that a greater freedom of choice does make patients happier on the whole, it could be argued that it is not the responsibility of publicly funded healthcare systems to help people feel good about matters that are not clearly related to their health.

A rather different, and perhaps more promising, way of legitimizing co-payment solutions within what we might call the autonomy framework would be to appeal to the liberal tenet that freedom of choice ought to be the *default*. The suggestion would be that one needs special reasons to restrict choice, given the general value of having a society incorporating individual freedoms. From this perspective, to enquire about what will be gained by specific new options introduced by a co-payment model is to ask the wrong question. Rather, the argument would go, if someone is prepared to offer additional healthcare options, for an additional levy, this should indeed be offered unless there are specific reasons not to do so. Anything else would be in conflict with fundamental liberal values.

Again, the distinction between permitting co-payment and facilitating it may be relevant here. If the main consideration is that increased freedom of choice will make people happier, just by virtue of having the choices, what is critical is at most that it is made known to people that co-payment is an option; there is no need to have them actually utilize the options introduced. The same point applies if the argument for co-payment solutions is that in a liberal society special reasons would be needed if choices are to be limited. If, on the other hand, the idea would be that it is patients actually utilizing the added options that justifies the greater freedom of choice – by perhaps helping them satisfy certain specific preferences – merely permitting this model may not be sufficient, since efforts may also have to be made to encourage, or otherwise help, patients to actually opt for the supplementary products or services.

On the matter of freedom of choice, will it matter what the new options, made available through co-payment, are, or how many they are? If the basis for offering greater freedom of choice is patient enjoyment, this is an open empirical matter. If, on the other hand, the argument is not so much that patients’ freedom of choice should be expanded, but rather the basic liberal contention that it should not be restricted, then the number of extra options offered and their putative value should be somewhat less relevant. The *clinical* value of the options introduced by co-payment will, of course, have bearing on whether the autonomy argument is powerful enough to show, or at least suggest, that co-payment ought to be allowed or even encouraged. If the additional treatment options promoting patient autonomy are agreed by medical experts to be significantly sub-optimal relative to those already offered to the patient, there will be independent reasons to conclude that society should not contribute to the funding of them. Most importantly, the offer of the additional treatments would violate principles of evidence-based medicine. It could also be regarded as something that is in conflict with the principle of beneficence (Beauchamp and Childress 2001), and hence also in potential conflict with professional codes of conduct.

Finally, while offers of potentially inferior treatments, and aids, and the like, would conflict with established prioritization principles in publicly funded healthcare, it should be stressed that the fact that co-payment options are possibly not *cost effective* does not weaken the autonomy argument. Certainly, the exclusion of those options from the public package may be based on the assessment that, although they are likely to help the patient, they are too expensive in relation to the predicted benefit.^4^ But considerations of cost effectiveness, as normally understood, will not be applicable in this case, precisely because society is not carrying the full cost and therefore will not need to assess whether this cost is justified by what is accomplished. The question for society is merely whether the public part of the cost is warranted. The patient who is paying for the extra options may believe the costs to him or her to be worthwhile.

To summarize, co-payment may deliver increased freedom of choice, but whether the values, or interests, served by this freedom will be promoted depends just on what those values or interests are, and on how, in detail, the model is employed. As was the case with the argument from health benefits, co-payment may still end up not being justified, all things considered. Most importantly, the anticipated gains in patient autonomy could turn out to be marginal, and as we shall contend, there is no shortage of arguments against co-payment.

### Quality and Safety of Care

Another potential advantage of co-payment is that it might serve to increase the transparency of, and control over, treatment or care that would otherwise be purchased entirely outside the public sector. Additions made available within the publicly funded healthcare systems may, for instance, strengthen certain quality and safety aspects of care. And having all of the treatment under the supervision of the same healthcare facility may ensure better continuity (Richards 2008). If co-payment for additions can be made in collaboration with the publicly funded healthcare system, patients will not need to take risks by buying and using private drugs, or by buying devices (or components for devices) without informing the healthcare personnel they ordinarily deal with. This might help patients to use their money wisely. It may also help those working in the public system to obtain a better picture of the patients’ overall care needs, and eventually provide scientists with high quality data which can benefit future patients who suffer from similar problems.

This supposed advantage of co-payment is, of course, heavily dependent on the way it is implemented into the healthcare sector. It also raises questions about how well the state, through its regulation and oversight, ensures that acceptable standards of safety, quality and transparency are met in the private healthcare market. As to the first point, there are certainly ways in which co-payment solutions could fail to secure the desired quality and control. In Sweden, for instance, sharp criticism has been directed at co-payment solutions where those in need of hearing aids receive vouchers that can be used to cover part of the cost of devices that fall outside of the funded collection (Hörselskadades riksförbund 2014). Patients are encouraged to purchase expensive devices that are arguably no better than those freely available.

As to the second point, the more tightly a private market is regulated, and the more that transparency is required, the less are the risks of lower quality of care. But tighter regulatory controls may also weaken the relative advantages of the co-payment system, in that it could be expected to limit the freedom of choice. Again, here, it is impossible to evaluate co-payment (or fully public or private models) as a financial model in abstraction from the particular (legal, organizational, cultural etc.) setting in which it is implemented.

## The Case Against Co-payment

Some commentators believe that a case can be made against allowing co-payment solutions to enter the publicly funded healthcare system (Smer 2015). Irrespective of how strong that case actually is, it is certainly true that a number of concerns arise. One worry is that co-payment will have unintended side effects that will in the end (indirectly) harm patients. Another is that co-payment is in conflict with the moral foundations of publicly funded healthcare. Shortcomings largely connected with current public healthcare systems will not be discussed below, unless they are crucial for understanding the challenges of introducing co-payment.

### Health Inequalities and Inequalities of Opportunity

While ideally co-payment for additional care will not lead to anyone becoming worse off, it may lead to an increase in health-related inequalities among citizens, since it allows some individuals to raise their level of health beyond that of some of their fellow citizens. Add-ons may simply be too expensive for some, who will have to be satisfied with treatment and care of the kind funded within the public healthcare system. Others, suffering from the same conditions, may be able to maintain better health by making use of their greater economic resources. Although this kind of health-related inequality would be unintended, it is nonetheless an effect that should be expected. After all, co-payment is about making options available to those willing and able to pay, and not everyone will be fortunate enough to fall into that category. To what extent, exactly, co-payment will *actually* increase health-related inequality is an open question. It depends, for example, on which co-paid solutions are allowed and what the alternatives are. For example, if co-payment is not introduced, might worse inequalities arise as a more select number of individuals enhance their level of health by choosing to opt for *private* health care, either inside the country or by travelling to another country? Inequalities resulting from such circumstances should be considered in evaluations of allowing co-payment for additional care in publicly funded healthcare systems. At any rate, the effects of the co-payment solution would have to be studied empirically, with an awareness, of course, that what those effects will be is going to depend, in part, on society’s ambition to influence what will happen.

The strength of the health inequalities argument against co-payment hinges not just on whether inequalities are likely to obtain if co-payment solutions are put in place, but also on whether these predicted inequalities have any, or any significant, normative bearing. It could, for instance, be countered that the health benefits within reach for some but not others under a co-payment arrangement are of such minor importance that any resulting inequality in those benefits is not seriously objectionable. After all, in the context we are considering the public healthcare system will still be committed to meeting citizen’s most important medical needs, and what the add-on buys the patient will secure only kinds or degrees of health benefit that seem comparatively less important. The less important the relevant utility, the more likely it is, surely, that inequalities in that utility can be countenanced. In the words of Harry Frankfurt, the ”egalitarian condemnation of inequality as inherently bad loses much of its force… when we recognize that those who are doing considerably worse than others may nonetheless be doing rather well” (Frankfurt 1997, 5). The stronger one’s commitment to liberalism, the more willing one will be to allow inequalities in health or opportunity as inevitable by-products of freedom of choice.

Equality is a complex notion and the initial impression that co-payment solutions may create morally unacceptable inequalities can also be challenged by looking more closely at underlying assumptions about *who* we are taking to have a legitimate demand for equality, and about the relevant *currency* of equality. Let us examine these assumptions in turn.

It is one thing to assess inequality between patients suffering from the same medical condition and another to assess inequalities between patient groups or between all citizens. Where, for example, a patient’s situation is compared with that of the whole population (average), rather than with that of persons suffering from the same condition, co-payment might serve to close the gap rather than widen it. In other words, co-payment may ensure that there is more equality between those who are ill, as a group, and those who are not, as a group, when it comes to health. From this perspective, it is not entirely clear how equality considerations bear on the issue of co-payment. As to the currency of equality, it could be argued that we need to remember that inequalities in health are not the only ones that matter. More specifically, in this particular case, it might be suggested that any increase in health inequality will be offset by a reduction in the inequality of economic resources, and that the latter will, in turn, introduce greater equality in other utilities that can be purchased.

It could be argued that the main problem is not (at least, directly) about *health* inequalities, but rather that the introduction of the co-payment model is difficult to square with the broadly agreed upon commitment to equality of *opportunity*. It is true that the goal of a publicly funded healthcare system, like the one that operates in Sweden, is to provide good healthcare for all in the society. However, this goal may be viewed as utopian, and certainly the public commitment could never be to guarantee equality of health. More realistic, perhaps—and a more obvious normative cornerstone in this kind of healthcare system —is a commitment to provide healthcare to all on equal terms. A common interpretation of this commitment, albeit not necessarily the only one, is that everyone should have access to care on the basis of need only (Smer 2015; Richards 2008; Weale and Clark 2010). 5,6 In other words, no special treatment should be given to some individuals if the very same treatment is denied to others in the same need. In Sweden, for example, this principle of equal opportunity is built into an ethics platform intended to guide decisions concerning healthcare priorities, which is accepted by the Swedish parliament (Statens offentliga utredningar 1995). Co-payment alternatives, on the other hand, are feasible only to those who are able to pay for them, and not all patients will be able to do so. Some patients who would consent to the treatment if it were freely available may decline it when it is presented as a part of a co-payment solution, either because, in absolute terms, they do not have the financial resources to pay the additional cost, or because doing so would have a significant negative impact on their personal economy. Their healthcare needs, however, could be the same as for those who can afford the additional cost of the co-payment offer.

Given that, for all practical purposes, the co-payment model offers only some individuals certain health benefits, irrespective of need, the model may indeed seem to violate this commonly accepted principle of equality. It could, however, be argued that this impression is misguided. After all, in publicly funded healthcare systems of the kind implemented in Sweden and the UK, the co-payment options will become available only if they fail to pass prioritization appraisals in which both health needs and cost effectiveness are assessed, both within and among different patient populations. This means that co-payment is compatible with patients’ needs being treated as equally important. Were healthcare professionals to give extra credits to those individuals who have paid for co-payment solutions, and regard them as first-class patients in various respects, with others being given lower priority, that would conflict with the principle of equal rights to healthcare. It is, however, an open question whether this will actually happen, and whether it can be dealt with by means of regulation and oversight.

Perhaps the strongest inequality-related argument against co-payment focuses on *perceived* inequalities rather than actual ones. For example, while from a theoretical perspective there are numerous ways of measuring inequality, people are typically more sensitive to some comparisons than others and will feel more negatively affected by society facilitating inequalities with respect to those. The fact, if it is a fact, that someone else, also suffering from cancer, has access to better treatment than one does oneself may well upset one’s egalitarian intuitions more than a system that permits broader population-level inequalities. And regardless of what actual inequalities there may be, co-payment can be viewed as a controversial *symbol* of inequality. Only some individuals will be able (or have the financial comfort) to purchase the additional care offered through co-payment. Given this, allowing co-payment might send an unfortunate signal that acknowledges and legitimizes the idea that humans are divided into groups based on their class or income. Certainly people are already divided along those lines in other areas in society. Not everyone can buy a new car or house, to mention just two examples. It is true that private treatment and care options are permitted in almost all welfare states, but they are typically not initiated and managed by state institutions in the way co-payment solutions may be. In this way, the public healthcare system would become an accomplice in the creation of (subjectively) objectionable inequality. Again, some of the other inequalities of opportunity that we already face do not involve serious health care needs of the kind that invite the idea that inequality is offensive. It is not far-fetched to assume that this symbolic inequality is precisely the kind of thing that people feel concerned about when confronted with the co-payment model. Co-payment may, in other words, function as a beacon that reminds us that wealth plays a crucial role even when we are talking about those in genuine need of care. As this concerns public attitudes, policy on co-payment models ought to be informed by empirical studies giving us a better understanding of what people’s opinions actually are, and what contributes to them.

### Further Problems Associated With The Implementation of Co-payments

Co-payment has often been characterized in a somewhat idealized manner, meaning that few of its potentially negative side effects have been discussed. In real life, however, major changes to the healthcare system may have significant consequences both for the system as such, for the healthcare professionals who work in it, and for patients. These consequences need not have anything to do with the underlying goals of the changes. They might be completely unintended. Healthcare is very complex. Many different factors will contribute to the actual effects of the introduction of a co-payment model, and plenty of things could go wrong.

Depending on the broader setting in which it is employed, co-payment could, for example, bring about conflicts between needs and demands (Smer 2015). The arrival of such a new market mechanism within the public system could have the unfortunate (and unintended) effect of displacing more highly prioritized healthcare resources. Co-payment might thus introduce changes to the system that will have a negative effect on groups with significant healthcare needs. While it could be argued that the co-payment model as such cannot be blamed for unjustified decisions not to include certain kinds of treatment, aids, and the like, in the public package, it bears repeating that this model cannot be evaluated in a vacuum: it must be assessed with one eye on conceivable implementation problems. Consideration must be given to whether society is committed to addressing those problems and devising plans that deal with them.

That the success of a co-payment model in part hinges on public perceptions has already been touched upon. In addition to whatever effects it may have on the public’s perception of inequalities in healthcare, the implementation of a co-payment solution may affect people’s outlook in other ways. It has been pointed out, for instance, that co-payment may induce stagnation in society’s public healthcare ambitions, and it has been cautioned that publicly funded healthcare could develop into a second-best alternative compared with privately funded alternatives (Smer 2015). Following such a development, trust in the public healthcare sector may be eroded and the taxpayers’ willingness to subsidise the sector may be weakened. Possibly, this willingness could also be weakened by people being expected to already pay for the health care they desire (through co-payment).

In the Swedish debate it has been suggested that co-payment solutions may lead to a risk of improper marketing. Users may come under pressure from caregivers to choose more advanced healthcare alternatives than they actually need. Several anecdotal examples of this have been described (Hörselskadades Riksförbund 2014). The co-payment model may evolve, for example, in such a way that it allows bonuses to be paid to those working with co-payment who achieve pre-defined levels of uptake. If kick-backs are indeed awarded to those who offer co-payment alternatives to patients, or are successful in selling those alternatives, real doubts may emerge about whether the options will be presented to patients in a neutral and accurate manner.

Some critics have argued that implementation of co-payment will lead to a rise in total healthcare spending in the affected countries (Bloor 2008; Weale and Clark 2010). Co-payment for additional care will not be cost-efficient, it is argued, since the healthcare measures in question will not have met the requirements set down by the UK’s National Institute for Health and Care Excellence (or similar institutions in other countries) with regard to cost-efficiency. In response to this it should be noted, as was pointed out previously in this article, that the additional costs for add-ons in a co-payment package come from sources outside the tax system. These costs should not, therefore, affect costs in the public healthcare system.

Finally, in the Swedish debate it has been pointed out that co-payment solutions may leave it unclear who is responsible for different parts of a co-payment package. This could be important if complications connected with the package occur at a later stage. This argument certainly has a bearing on other systems where public and private spending are mixed, such as those parallel public-private systems that currently exist in the above-mentioned countries, and in most other countries as well. Whether the worries are exacerbated in a co-payment system may depend, to an extent, on how extensively the public healthcare organization will be involved in administration of the added products or services, and how the relevant responsibilities are perceived by those who are considering utilizing the additional options. Conceivably, the more engaged the public healthcare organization is in facilitating co-payment offers, the greater the risk of unclear responsibilities and accountability.

Most of the problems described above are fairly speculative, and some of them are perhaps best described merely as potential risks. As has already been stressed, to the extent that managing such risks is under decision makers’ control, arguments for or against co-payment should not be made without taking into consideration how society intends to implement the model. But whether these risks are ultimately considered slim or significant, it should be noted that they cannot be avoided altogether. Precaution seems advisable when the decision at hand – in this case, whether to introduce the co-payment model – is optional. Arguably, the better the current system works, the more problematic it becomes to expose patients and other stakeholders to risks that can be avoided. And given the uncertainties involved, if the model is unlikely to satisfy important healthcare needs, the case for (at least) facilitating co-payment seems weak.

## Concluding Remarks

Financial constraints challenge publicly funded healthcare systems in many ways. One unavoidable consequence is that patients are occasionally denied certain kinds of treatment or care. The reason for this denial may be that the treatments are regarded as too expensive or not cost-effective enough to qualify as standard care. Co-payment solutions would however make such health care available to patients.

Many of the ethical consideration regarding co-payment revolve around the effects of this model on, among other things, population health, freedom of choice, patient safety, equality, and public trust in the healthcare system. Determining what those effects might be, would require the model to be studied empirically. Ultimately, those effects would have to be compared to the effects of not introducing co-payment solutions. While perhaps feasible, doing this in a methodologically reliable way would be very difficult.

Saying that co-payment models would have to be studied empirically, in order for their effects to be reliably evaluated, does not equate to saying that such models ought to be introduced and empirically assessed. As has been indicated, there are strong reasons in favour of a precautionary approach. Co-payment for additional care enters the picture after priorities have been set, which means that treatments for significant healthcare needs at reasonable cost should already have been assured. Given this, co-payment ought to play a minor part in overall healthcare. Hence, the opportunity cost of waiting before introducing the model is likely to be fairly small – at least, across the health sector as a whole. Given the risks of allowing co-payment, one should proceed carefully.

Clearly, as soon as co-payment solutions are seriously considered, policy questions move to centre-stage. The policies will need to indicate where the line is to be drawn between what can, and cannot, be allowed to enter the system in the way of treatments and care. They will also need to make it clear how co-payment solutions should be assessed and monitored. Further research is needed not only on the implications of theories of distributive justice on the ethical justification of co-payment solutions, but also on the regulatory challenges and prospects of dealing with the whole spectrum of concerns associated with implementation of co-payment solutions in specific healthcare, political and organizational settings.

## Footnotes

1 Other terminologies have been used to refer to this model (Mossialos et al. 2015). While some writers use the term “cost sharing” (Barnieh et al. 2014) others refer to “excess charges”, “additional services”, and “top-ups” (Richards 2008).

2 In group-based systems certain population groups are eligible for publicly funded care; in sectorial systems some healthcare sectors are publicly financed. Different versions of these models have been incorporated in healthcare systems in different countries (Tuohy et al. 2004).

3 In October 2015 a senior consultant at the department of lung diseases in Haukeland University Hospital, Norway, applied to the local board of ethics (Notat fra Klinisk etikk-komité, Helse Bergen 2015) to use a co-payment solution involving Nivolumab (Opdivo), a drug that is given intravenously for the treatment of advanced cancers. The consultant had found that some patients with lung cancer were willing to pay the extra cost for Nivolumab if the drug could be administered at the hospital in conjunction with planned visits and other funded healthcare measures relating to the monitoring of the disease. At the time in Norway, Nivolumab was not a publicly funded form of treatment for lung cancer, although the evidence suggested that it promoted an extra median survival period of three months for patients with this diagnosis.

4 It has been pointed out that the “position of charging for treatments that have not been deemed as cost effective” might be regarded as perverse (Richards 2008). Such an impression, however, relies on a misunderstanding, since almost any positive effect may seem worthwhile at the individual level. Under no circumstances should we apply societal principles of prioritization, which are constructed in order to prioritize between groups, at the individual level.

5 In the aforementioned evaluation of a potential co-payment solution involving the drug Nivolumab the local board of ethics in Bergen, Norway, (Notat fra Klinisk etikk-komité, Helse Bergen 2015) referred to a principle of equity in healthcare that should not be violated. On the boards’ interpretation of it, the principle means that equals should be treated equally.

6 Matching intentions can be tracked to international statements about Universal Health Coverage (UHC). UHC is defined by WHO and the World Bank as follows: “all people receive the health services they need without suffering financial hardship when paying for them” (Lancet 2016; Norheim 2015; Chalkidou et al. 2016).
